# Impact of Process Variables on Part Quality in Progressive Stamping

**DOI:** 10.3390/ma19020312

**Published:** 2026-01-13

**Authors:** Juras Skardžius, Justinas Gargasas

**Affiliations:** Department of Mechanical and Materials Engineering, Faculty of Mechanics, Vilnius Gediminas Technical University, Saulėtekio al. 11, LT-10223 Vilnius, Lithuania; juras.skardzius@vilniustech.lt

**Keywords:** progressive stamping, process variables, die clearance, tool wear, lubrication, bending angle, part quality, statistical analysis, sensor monitoring

## Abstract

The progressive stamping process includes blanking, piercing, bending, and drawing operations on press machines with a single die set for high production runs. The processing conditions at individual progressive stamping stations are intricately coupled, posing a challenge for maintaining part quality at high production rates and dimensional precision. This study investigated the effects of the die bottom dead center (and later, BDC) depth, punch-die clearance, tool wear condition, and lubrication performance on the precision of stamped parts and bending angles. Quality characteristics were measured using a coordinate measuring machine (CMM) by employing a thin-sheet steel progressive die in a factorial experimental design. Using Pareto effect plots and the MINITAB platform, it was observed that for part bending angles, the first greatest factor of importance is BDC, followed by clearance as the second greatest, and then tool condition. The results reveal that although it affects part quality through interactions, the lubrication effect is not as significant as the main factors. However, SEM analyses show that worn tools and inadequate lubrication induce grain boundary separation, microcracking, and dislocations, while proper lubrication and sharp tooling maintain more homogeneous grain structures. Research indicates that achieving the full control of part quality in the progressive stamping process requires more than bottom dead center (BDC) adjustment; factors such as component clearances, punch condition, and lubrication level must also be considered. Process-based knowledge of the relationships among process parameters in multi-stage stamping processes can be used to develop adaptive monitoring systems that stabilize part geometry and minimize production variation.

## 1. Introduction

Bending is one of the most fundamental stamping operations, but that does not mean it is easy. Because of the elastic rebound nature of materials, test repeatability can be high, leading to significant variability; therefore, it needs to be controlled and adjusted frequently. It is generally believed in the production industry that the bending angle can be changed by adjusting the tool sliding fit level. However, this assumption is not fully true because the impact of other process parameters on the bending angle needs to be demonstrated. To this end, the combination of different forming, cutting, and trimming operations is performed sequentially by strip-feeding stages. The press makes only one stroke to advance the blanks through several stations, which perform one or more operations such as blanking, piercing, bending, drawing, coining, and trimming at different heights or at different time intervals until a finished part leaves the tool. The workstations create strong interdependencies among process variables, as they influence one another in the production series. According to Molitor et al. [1], all steps in the multi-stage chain must be examined to identify productivity-inhibiting factors, such as friction, vibration, or misalignment. Dwi et al. [2] conducted a recent systematic review analyzing the impact of progressive die design and process integration on quality and productivity. Some researchers, such as Farioli et al. [3] and Dwi et al. [2], have recently provided an overview of progressive die design and the effects of process integration on productivity and quality. Trade-offs are endemic in progressive stamping: designers can design useful but conservative stroke rates that maintain stability and tool lifetime; however, this restrains the throughput, resulting in non-ideal part quality (e.g., inhomogeneous strain distribution or surface defects) when faster stroke rates are forced upon it (Molitor et al. [1]). Therefore, part quality for progressive part stamping is a function not only of station-by-station design but also of how the process variables are transferred in sequence.

Punch and die clearance, cutting edge radius, die corner radius, punch tilt, and die shape are “normally” critical to overall part quality. According to Rizak J. et al. [4], cut-edge properties (rollover, burnish, and fracture zones), burr height, cutting force, tool wear, and dimensional accuracies for blanking are significantly affected by radial clearance in blanking and piercing. In [1], the authors show, via finite element simulation, that decreasing clearance increases the peak punch force and cut-edge quality but at the cost of increased stress and frictional contacts. Mucha et al. [5] studied the effects of clearance and tool edge inclination on pressure distributions and punch loading and reported that the selection of inclination angle, as well as clearance values, leads to a reduction in burr formation and an enhancement in deformation loads. In blanking, Chan et al. [6] show, with an experimental ANOVA (Analysis of Variance) study, that tool wear radius is approximately twice as influential as clearance in burr height control [6]. Earlier work from these authors also demonstrated that a clearance of around 10% of the sheet thickness could make blanking force minimal, while lower (~5%) clearances led to incremental minimization of the fracture angle, but wear resistance varied with the choice of clearance [7]. In bending and forming, the ratio of the die radius to the workpiece sheet thickness plays a critical role in controlling local stresses and elastic recovery (springback). Strip layout, station order, stripper and pilot design, and sensor integration need to be considered when working on progressive die design. The die layout defines strip flow, material feed, and alignment tolerances, which cause quality fluctuations. Tool wear has a noticeable impact in high-volume stamping operations, where continuous wear is associated with variations in geometry, changes in clearances, increased friction, and loss of quality [8]. In current surveys, wear mechanisms (abrasive, adhesive, fatigue, and tri-bo-oxidation) and relief strategies (surface coatings, texturing, lubricant strategies, and tool material selection) in stamping conditions are classified. Researchers Kubik et al. [9] studied wear-detection techniques in both roll forming and stamping conditions, including sensor-based monitoring to detect wear before quality drops. In the area of progressive stamping, Farioli et al. [3] propose a data-driven tool-failure prevention approach using global and local force sensors to identify unhealthy tool conditions, which can lead to catastrophic failures that affect part quality. Molitor D. A. et al. [1] apply deep convolutional neural networks to workpiece images for the classification of the tool wear stage in the blanking process, relating wear development to product trends. Researchers Molitora et al. [10] and Schlegel et al. [11] apply semantic segmentation networks (e.g., U-Net) to segment the worn area on tool surfaces at high stroke rates to quantify geometric changes due to wear. Accordingly, the inclusion of tool health monitoring into progressive die applications is a potential path towards quality assurance over large production runs.

Dimensional control in progressive stamping is critical to maintaining part quality, repeatability, and functional reliability in high-volume production. This study demonstrates that process variables such as die BDC level, punch-die clearance, and tool condition interactively govern part geometry, springback, and microstructural integrity. Precise control of these parameters ensures uniform material flow, minimizes elastic recovery, and reduces defects like thinning, microcracking, and grain boundary separation. In industry, this translates to higher yield, reduced scrap, improved surface finish, and longer tool life. Consequently, robust dimensional control is not just about achieving target bending angles—it underpins overall process stability, part performance, and cost-effective production in multi-stage stamping operations.

The mechanical properties of the sheet (yield strength, work-hardening exponent, anisotropy coefficients, and strain-rate sensitivity) significantly influence formability, springback, thinning, and defect susceptibility during forming processes. Thickness is a significant design variable: thinner sheets are more susceptible to bending strains and springback. Thickness variations (even within a coil) will lead to variations in local stresses and thus quality inconsistencies. Farioli et al. [3] and G.M. Sayeed Ahmed et al. [12] have experimentally measured springback in mild steel across thickness ranges and concluded that the deviation of nominal bend angle is proportional to thickness variation. They found that in progressive stamping processes, lot-to-lot mechanical property variation (due to variations in coils, residual stresses, or microstructural inhomogeneity) has been identified as a source of drift in part quality and the need for adaptive compensation. Interface friction between tools and sheet (punch, die, blank holder, stripper) governs metal flow, local stress states, heat generation, and the risk of galling or sticking, all of which affect final geometry, surface finish, and tool life. Chandrasekharan et al. [13] evaluated stamping lubricants experimentally across different temperatures, demonstrating that the proper use of lubricants decreases friction, lowers tool wear, and enhances the draw-in behavior of the material. In dry-stamping conditions or with high-strength steels, friction variation becomes increasingly vital (Folle et al. [14]). Friction is typically parameterized in simulation analysis by a Coulomb friction coefficient (or more complex friction laws), and sensitivity studies show that comparatively modest changes in friction coefficients shift strain distributions and alter springback predictions (Mucha et al. [5]). Moreover, lubricant quality can deteriorate over long production runs (due to breakdown, contamination, or temperature), leading to variations in local friction and thus quality drift. Monitoring friction trends is a practical monitoring problem in real stamping lines. Also, lubrication effectiveness may deteriorate over long production runs (due to breakdown, contamination, or temperature), leading to changes in local friction and, in turn, quality drift. Monitoring trends in friction evolution is a typical monitoring challenge for stamping companies.

One of the most noticeable and widespread quality issues in sheet metal forming is springback: the elastic recovery of the formed part after the removal of tooling loads. Springback causes deviations from intended geometry and usually requires overbending or die compensation [15,16,17,18]. Chandrasekharan et al. [13] provide a review of springback and control parameters such as material properties, friction, tooling geometry, bend radius, blank holder, and bending sequences. Lal et al. [7] studied experimentally and analytically the influence of sheet properties, bend radius, and process conditions on springback magnitude. Ahmed et al. [12] validated springback tendencies for different mild steels and measured average deviations. As progressive stamping often involves sequential forming or bending stations, springback interaction can propagate residual stresses of a previous station and can affect subsequent forming and the final geometry [19]. Compensation, therefore, must be dealt with in a staged (feed-forward or feedback) manner. In stretching, bending, and localized forming (such as deep drawing), nonuniform strain distributions cause thinning (material weakening) or localized tearing. Strain path development is dependent on process variables (blank-holder force, draw bead height, lubricant, die geometry). The research of Guo F. et al. [16] discussed multicriteria optimization, such as using Taguchi, Response Surface Methodology (RSM), genetic algorithms, etc., and applications in designing the stamping tools to achieve optimum results with minimum thinning, springback, and other defects, respectively. Some studies focus on the formation of wrinkles (compressive instability) during forming, noting that material flow control is crucial to avoid buckling [6,10,12]. Local failure criteria (e.g., ductile fracture models and stress triaxiality-based criteria) are commonly incorporated into FEM-based stamping simulations to predict tearing or necking sites. These predictions for growth are influenced by boundary conditions, friction, and local thickness variations.

Dynamic effects (material inertia, vibration, and resonance), even with high strokes, may decrease control as well as misregister the tooling, causing strip flutter, which results in dimensional variation [1,20]. Underlining that the interruption of throughput due to stroke-rate dependent anomalies, such as vibration or shift friction loss, is a further limit in comparison with quality. In multi-stage forming, sensor-based monitoring identifies anomalies at individual stations (e.g., force signal deviation, local lag). Schlegel et al. [11] examined a connection between force and bending signals and proved that feed-forward control is possible. In the field of progressive die design, Dwi et al. [2] also note that dynamic-induced defects can be addressed through innovations such as AI for anomaly detection and CAD-based generative die layouts.

The literature and the study indicate that process variables in progressive stamping are highly interdependent, and optimizing one factor in isolation (e.g., die BDC) is insufficient. Comprehensive, multi-factorial approaches combining geometric control, tribology management, tool wear monitoring, and adaptive strategies are required to ensure consistent part quality both at the macro (geometry) and micro (grain structure, fatigue resistance) levels.

The main aim of this research is to investigate the impact of progressive stamping process variables on part geometry, analyze the interactions between process variables, evaluate microstructure, and determine the existing mechanical integrity of materials.

## 2. Experimental Research Methodology

### 2.1. Process Design

**Figure 1:**
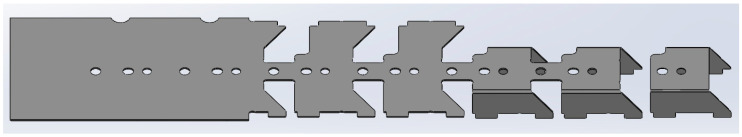
Research object strip.

Factor analysis allows for the simultaneous study of the influence of several process parameters (factors) and their mutual interactions on the selected characteristic. This is an important advantage of this method compared to conventional methods of analysis, where only one variable is changed and its subsequent effect is observed. Unfortunately, increasing the number of parameters rapidly increases the number of tests required and makes the analysis more complicated. Therefore, it is important to study only the essential factors that can be controlled during production. As a research object, a progressive tool with seven workstations was selected (Figure 1) and designed to be compatible with S355MC material. For tests, as in standard production, material of controlled thickness (±0.1 mm) was used, with the mechanical properties presented in Table 1.

One such parameter is the previously mentioned BDC of the die, which is controlled by adjusting the position of the press upper plate. By lowering the slide, the bending punch’s travel increases, pressing the material more. In this way, the angle change due to the material’s elastic recovery is compensated.

**Figure 2:**
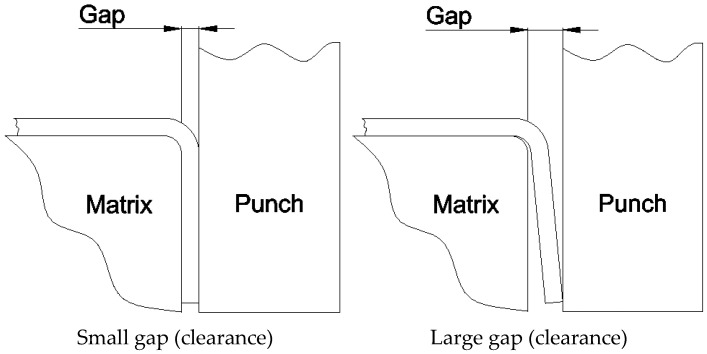
The influence of the gap between the die and the punch on the bending angle.

Another essential parameter is the clearance (gap) between the bending die, workpiece, and the punch. When the material is thin, there is a larger gap between the bending die and the punch, so the material is not bent all the way, and when the material is thicker, the gap is reduced, so the material is folded more (see Figure 2). Finally, when the clearance becomes negative, the material is crushed. For these reasons, the gap is an important parameter in the stamping process and can also affect the bending angle, so it must be adjusted based on the material thickness and other factors.

The third important parameter is the condition of the bending punches. Over time, these parts wear, which changes their geometry and contact surfaces and increases the clearance between the punch, die, and the material, as well as increases the friction between them. All this inevitably affects the bending process, so tool wear must be controlled by timely replacement and sharpening of dies and punches.

The fourth parameter, which we decided to include in the analysis, is lubrication. The oil reduces friction between the contacting surfaces, so the bending punch slides more easily along the material’s surface. As a result, the wear processes, including abrasive wear and punching, which dominate during bending, are slowed down. For these reasons, we believe that lubrication efficiency can also significantly affect the bending angle.

The rest of the process parameters, such as production speed, material feed length, etc., generally have less influence on bending or require specific conditions for their effects to become noticeable. For the mentioned reason, it was decided to ignore them.

After identifying the essential parameters of the process under study, we selected their high (+) and low (−) levels. These are summarized in Table 2.

The optimal BDC was obtained at a slide position of 381.1 mm. By allowing the slide to lower below this limit, part would be unnecessarily over-pressed. To obtain a low BDC point, it was decided to raise the slide 300 µm above the optimal position.

Unlike the BDC, changing the clearance is not so easy. This would have required repositioning the bending punches or adjusting their geometry. In both cases, experimenting would have made it difficult or impossible to return to the original position. Therefore, instead, it was decided to vary the clearance by using materials of different thicknesses: 1.0 and 0.8 mm. As the workpiece thickness decreases, its BDC decreases as well. For this reason, whenever working with 0.8 mm thick material, the slide was lowered by 200 µm to maintain the same compression level.

Changing the punch’s wear levels was even more challenging because wear is a progressive, irreversible process. In addition, modern punches can usually withstand tens of thousands of cycles before needing to be restored or replaced. For these reasons, it was decided to make a special punch with a R0.1 mm radius on the working edge to simulate wear.

Compared to the parameters discussed above, it was easy to change the lubrication intensity. For lubrication, the Rhenus FU60 emulsion was used at a 1:1 mixing ratio, with a fresh preparation pH of 9.0. The emulsion amount used, as per the tool production instructions, was considered high. Lower lubrication intensity was not difficult. However, in order to maximize the possible effect, it was decided to turn off lubrication. Also, each time lubrication was shut off, a few cycles were performed, the results of which were not considered to get rid of all the emulsion left between the bending punches and the workpiece.

After defining the high and low levels of the essential bending process parameters, all possible combinations were generated. These are provided in Table 3. To avoid random values, it was decided to produce five parts for each combination. Ideally, this should be done in a completely random sequence: choose a random combination, make one part, then choose another random combination, make another part, and so on until the parts for each parameter combination have been made. Then, the second and subsequent cycles should be started, with sequences that are also random and different from the first. Unfortunately, for practical reasons, this rule was not strictly followed.

### 2.2. Measurement Procedures

With all experiment cases, test conditions were tried to replicate as closely as possible—the selected production speed was 40 cycles per minute, material was taken from the same coil, emulsion was sprayed at the same concentration as in the specific working instruction, all the settings were changed, and the parts were produced by the same experienced operator. Because of the adjustment that had to be performed with the tool, the experiment took a couple of days to finish. Both the first- and second-day tests were evaluated as separate groups. The results were evaluated using the statistical software MINITAB 2.0. Each day’s results group was called a block in the program. The results were evaluated based on the Pareto principle. The Pareto principle is an empirical heuristic stating that, in complex systems, a relatively small proportion of factors accounts for a disproportionately large share of the observed outcomes. It suggests that approximately 20% of the inputs, variables, or causes are responsible for about 80% of the effects. Although the exact ratio is not universal, the principle highlights systemic imbalance and concentration of influence within datasets. In research data analysis, the Pareto principle is employed to identify the “vital few” contributors that exert the greatest impact on a given variable.

**Figure 3:**
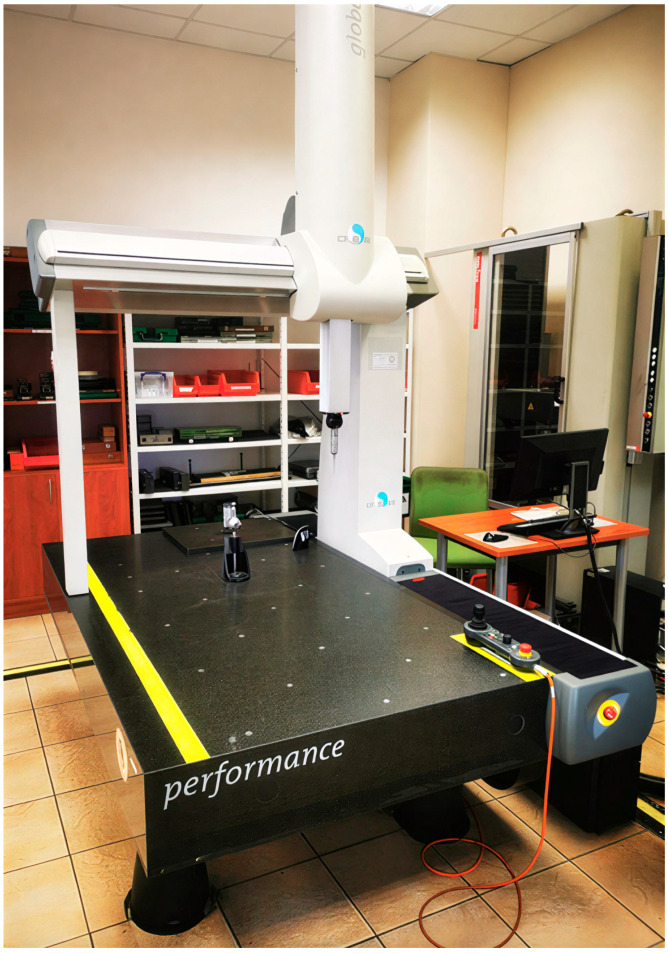
Coordinate measurement machine HEXAGON DEA Global Performance.

**Figure 4:**
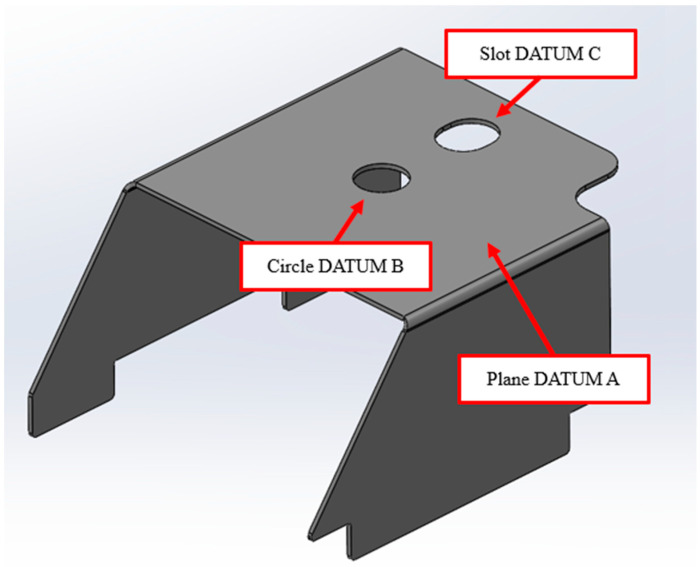
Measured part alignment scheme.

**Figure 5:**
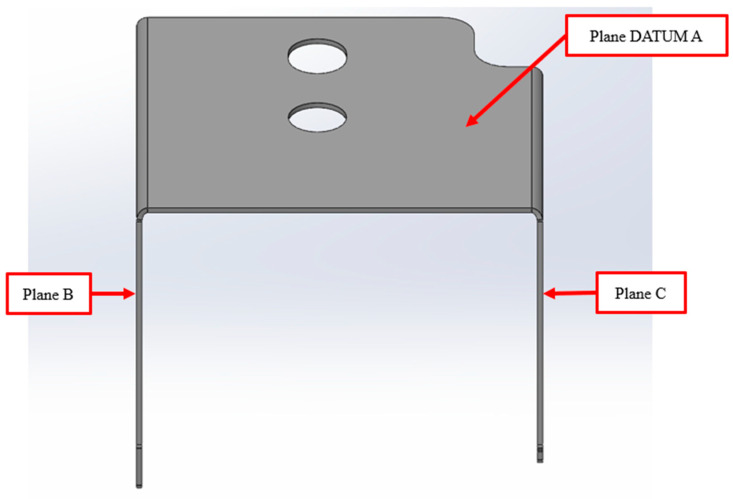
Measured part planes.

Collected samples were measured with the coordinate measurement machine (CMM)—HEXAGON DEA Global Performance (Figure 3). Part alignment in CMM is presented in Figure 4. The sample’s outside dimensions were 39.6 mm in width, 54.2 mm in length, and 33.1 mm in height. Plane Datum A was measured with 25 points; both circle Datum B and slot Datum C were measured with 15 points. The influence of process variables was expressed by the part’s bending angles from plane Datum A to plane B (angle AB) and plane C (angle AC) (see Figure 5). Each plane (B and C) was measured by 20 points. The CMM machine has an inherent systematic measurement error of ±4.5 µm. In addition to this intrinsic uncertainty, the geometry of the measured part and the limited number and distribution of measurement points influence the evaluation. As a result, the calculated angle between the two planes is subject to a potential angular deviation of ±0.003°.

**Figure 6:**
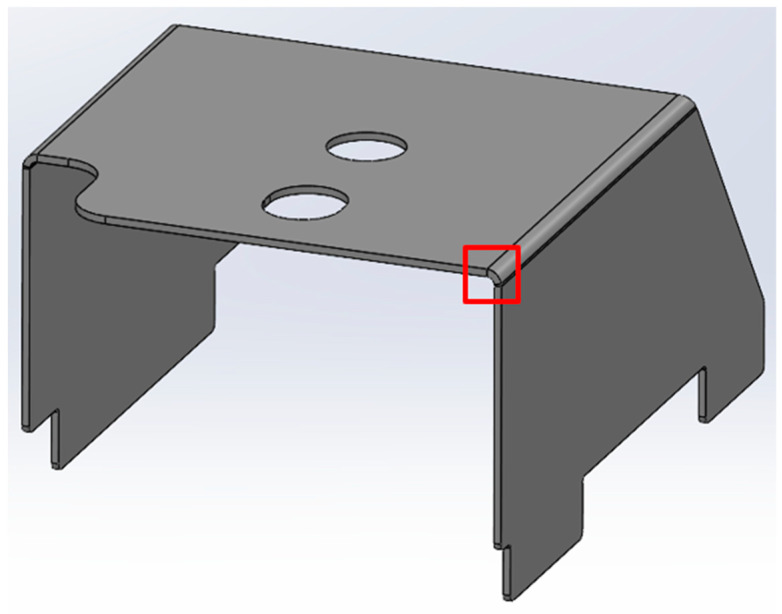
Measured area with SEM.

Later, selected samples with different factor combinations were examined by scanning electron microscope (SEM). The main purpose of the SEM microscope is to obtain high-resolution images, with a maximum resolution of 10 nm, which allows a deeper look into inner structure changes, material grain changes, and defect effects. Cross-sectional images of the sample are created during the imaging process by continuously capturing a series of small-region sample images with a 15% to 40% overlap. As samples are from ferromagnetic material, the magnetic field generated by electron flow and its delay can be used to examine and compare the material’s composition, allowing a deeper look at the overall effect of research variables. To assess the impact of process variables on the inner part structure and quality, the right back corner of the part (angle AB) was selected (Figure 6).

## 3. Results and Discussion

### 3.1. Effects of Process Parameters on Dimensional Precision

**Figure 7:**
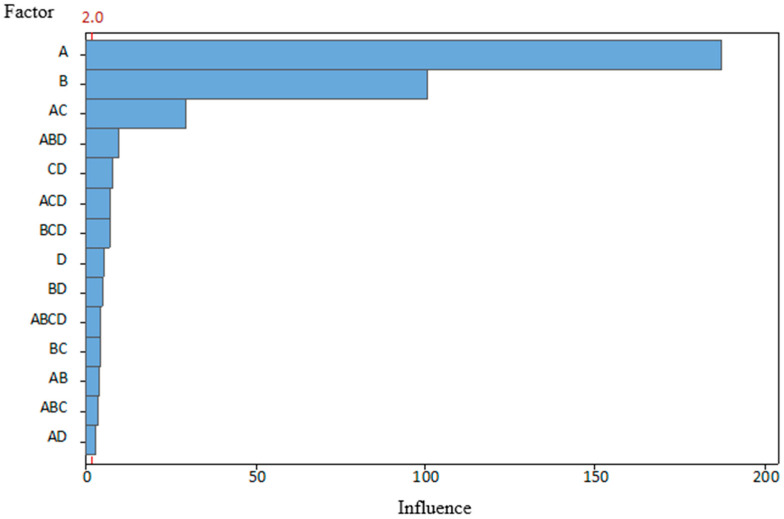
Pareto analysis of the influence of the studied parameters on the angle AB, when α = 0.05.

**Figure 8:**
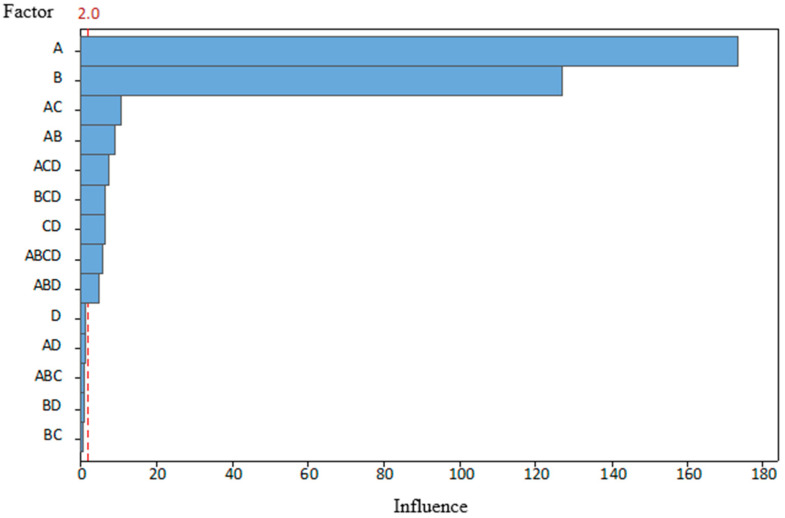
Pareto analysis of the influence of the studied parameters on the angle AC, when α = 0.05.

The tests were performed over 2 days; eight combination tests were conducted on both days. To avoid the influence of the previous combination and the introduction of additional errors, the tool was carefully disassembled and cleaned after each test. The order of the combinations was selected randomly using a program of random number generator, except that the first day was spent on combinations with a new punch condition and the second day on worn ones. Such an approach was selected to eliminate the possibility of incorrect assembly of damaged fixing elements, which could introduce additional errors. A total of 300 parts were produced for each combination. The last five parts were marked and set aside for CMM measurements and further examination. Test data and the results are presented in Table 4. After processing the data using the statistical program Minitab 2.0, the results are shown in Figure 7 and Figure 8.

The graphs clearly show that both the AB and AC angles were most affected by BDC (A), which partially supports our hypothesis. On the other hand, it is obvious that there are other significant factors. This influence of clearance (B), which, on the angle AC, is not far behind the BDC. The magnitude of angle AB was determined less by clearance, but its influence was still the second most significant. In addition, clearance can be observed in interactions with other factors. The condition of the punch (C) is not a separate factor for either corner, because, as previously mentioned, tests with new punches were taken on the first day and tests with worn out punches on the second day. As a result, it is not possible to answer unequivocally whether the possible effect is the condition of the punches or the influence of other factors that have changed during that time. This does not mean that wear does not affect the bend angle. The graphs presented show that its interaction with BDC is the third most important factor in determining the magnitudes of both angles.

Statistical results for the combination with BDC (A) and clearance (B) indicate less influence on both angles, because these process variables, by themselves, have the greatest effect. The lubrication (D) effect, on the other hand, shows the least impact on part quality, even though it is well known that without lubrication, heat and wear levels rise throughout the production process. This lack of effect can be explained by the fact that, although lubrication was turned off, the material coil (workpiece) itself had a small film of oil that acted as partial lubrication during the test. This bias may have led to a lack of statistical significance.

**Figure 9:**
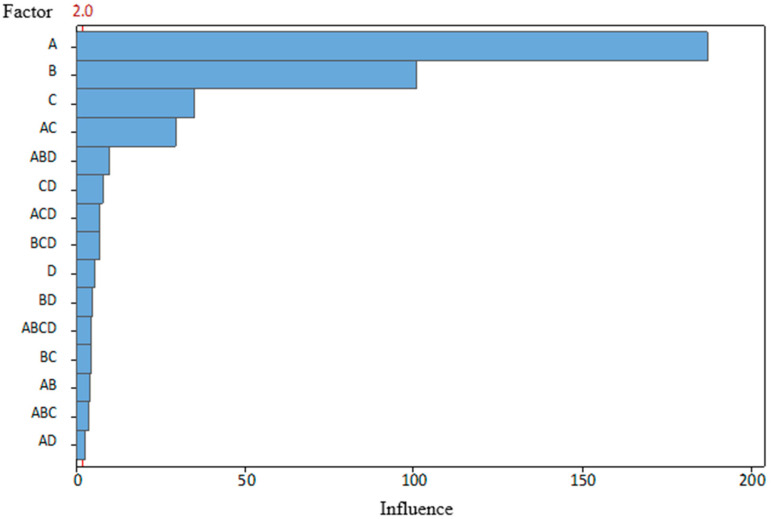
Pareto analysis of the influence of the studied parameters on the angle AB, when α = 0.05, and groups combined.

**Figure 10:**
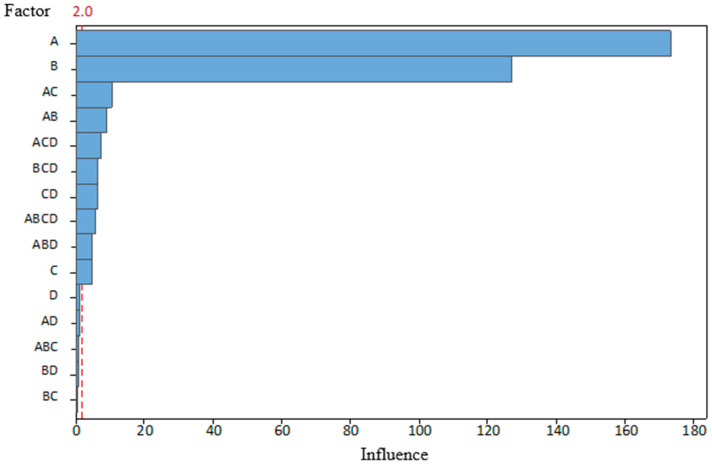
Pareto analysis of the influence of the studied parameters on the angle AC, when α = 0.05, and groups combined.

Moreover, it is also seen in interaction with other parameters. Therefore, it was decided to analyze how things would look if the assumption were made that the conditions did not differ significantly between the two test days, thereby allowing the two datasets to be merged into one. The results are presented in Figure 9 and Figure 10.

If the assumption is taken that during both test days, conditions were kept the same when working with new and worn punches, then the influence of the condition of the punches (C) would be the third most important effect for the angle AB. The effect on angle AC would be smaller but still statistically significant.

Returning to the previous discussion of the influence of process parameters, it was noted that lubrication had a small but still significant influence on the angle AB, and its effect on the angle AC was statistically insignificant—it is beyond the limit marked by the red dotted line. Such a difference could also arise because the bending line distances are uneven. However, it should be noted that lubrication interacted with other process parameters and affected both bending angles, so it cannot be considered completely insignificant.

Another important observation is that, for angle AB, the influence of any parameter or their possible interactions cannot be ruled out, which is quite unusual. For angle AC, only the effect of lubrication and its interaction with BDC (A) and clearance (B), as well as the interaction of clearance, punch condition (C), and the triple interaction of BDC (A), clearance (B), and punch condition (C), were statistically insignificant. It should be emphasized that the boundary between significant and non-significant effects was calculated using the traditional α = 0.05 level of significance. Changing this setting could cause the results to look slightly different.

Collected data show that although BDC is the most important factor in determining the angles obtained during bending, it is not the only important parameter in the process. Therefore, merely by adjusting the stripper’s position, the desired detail quality cannot be achieved. This requires additional control of at least the clearance (B) between the workpiece, die, and punch. Tool wear (C) must also be controlled. However, as mentioned, modern punches can perform tens of thousands of bending operations before wearing out, so this will not necessarily be relevant in everyday die tuning. An unequivocal answer regarding the influence of lubrication (D) was not met. On the one hand, the collected data does not show an unequivocal influence on the angle size. Lubrication, on the other hand, is important for the surface quality of the part. Therefore, based on experience, the use of lubrication during bending makes sense.

### 3.2. Effects of Process Parameters on Mechanical and Microstructural Quality, the SEM Observations

**Figure 11:**
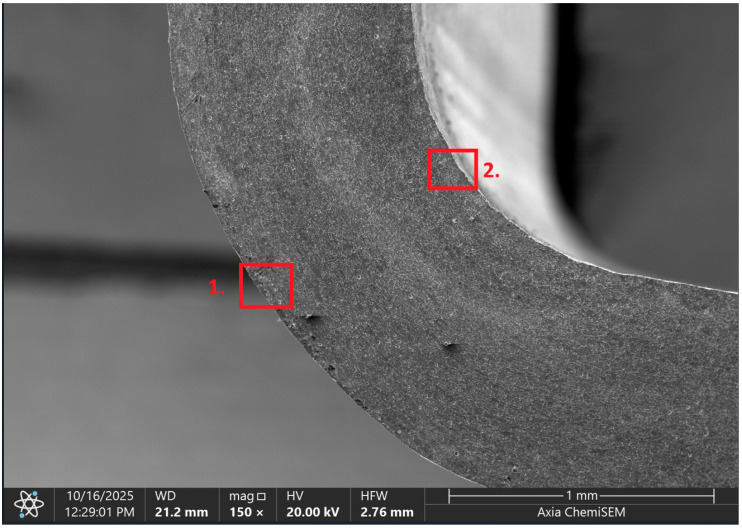
Material inner structure comparison places: 1—outer radius; 2—inner radius.

The selected samples used for Combinations No. 4, 6, 13, and 14 were analyzed by scanning electron microscopy (SEM) after dimensional measurements and statistical analysis. These combinations were selected for different process conditions, with regard to the BDC level, clearance, tool condition, and lubrication. These tribological conditions play an essential role in maintaining material integrity and preventing subsurface microstructural damage, even though direct tribological measurements were not performed. A qualitative comparison of process parameters in Combination No. 4, where all process parameters were set to their optimal levels, served as the reference condition. The outer radius (tensile side) and inner radius (compressive side) at the center near bend AB were separately analyzed by SEM (Figure 10). The first aim of this analysis was to qualitatively examine the variation in the surface and near-surface morphology during the process. Materials were collected without metallographic etching, and magnifications ranged up to 2500×. Areas tested by the SEM are presented in Figure 11, and the scanning results are presented in Table 5. Note that, under these preparation and imaging conditions, SEM analysis does not permit direct, reliable visualization of crystallographic grain boundaries, dislocation structures, or grain-scale deformation mechanisms. Accordingly, the current investigation is confined to observable morphological parameters, including surface roughness, shear markings, micro-voids, localized cracking, and near-surface deformational heterogeneity. Interpretation of bulk microstructural evolution or deformation mechanisms of such structures would need further metallographic preparation and higher-resolution techniques.

Material comparison shows a relatively, nearly constant and continuous surface shape (surface morphology) on the outer and inner bending radii. The surface topography was smoother, with fewer substantial shear marks or localized surface discontinuities on Combination No. 4 (reference condition). No persistent cracking networks, nor large surface voids, were present. The inner radius surface showed good homogeneity in deformation characteristics, indicating that the solid material flowed under compressive loading. In general, a relatively uniform microstructure of the formation medium for Combination No. 4 was observed under SEM, indicating that surface destruction and local deformations were kept at a low level under the process conditions.

In the fourth combination, the surface structure is generally very homogeneous and dense with low boundary relief and intergranular separation. Individual grains are not turned into circles, indicating the uniform strain distribution under the tensile load applied on the outer surface. Only small spots of boundary opening close to the edge, which may be driven by local surface tension relaxation or polishing artefacts, could be found. Some localized globular-like depositions can be seen in the sample. Probably these are the oxide or debris at the stamping surface, and there is no continuous cracking network observed. Further analysis of the inner radius shows that, under compressive stress, grains undergo mild bending and slight flattening towards the bend interface. Boundaries are coherent, and no obvious serious buckling or micro-void can be observed. The micrograph reveals that when all process parameters are appropriate, the material exhibits structural continuity along the bent portion, indicating that the adopted forming conditions avoid microdamage. It can be concluded that combination No. 4 is that against which all other combinations are compared in terms of strain distribution and tensile–compressive stress balance; no large opening of grain boundaries or initiation of microcracks could be observed, suggesting that frictional and residual-stress effects were well maintained.

The effect of disabled lubrication, as well as BDC (Combination No. 6), resulted in a more varied surface type than the reference sample. On the outer radius side, elongated surface features and bending-aligned shear marks were more common. Localized surface tearing and fine discontinuities near the tool–material contact interface indicated that surface-level deformation had increased. The surface features of the inner radius showed irregularity and focal regions of intensified deformation. These observations indicate enhanced frictional interaction and nonuniform material flow during bending. Immediately after treatment with a single gage, the microstructural damage mechanism, lacking distinct characteristics, was demonstrated in the present SEM images, while the surface irregularities imply degradation of surface integrity relative to the reference condition.

Analysis of the inner radius reveals strong shear bands and undulating boundaries in the microstructure of the compression zone. A few boundary crossings exhibit small gaps and darker contrast dashed regions that are associated with microdelamination or incipient decohesion. The wide, enveloping strip along the die contact surface suggests material accentuation and perhaps even some sticking to the tooling face. The densities and the roughness of the grain boundaries are larger than those in the reference, indicating that it was not homogeneous compression of the material, and there was some non-sticking or sticking–sliding on part of these regions. Combination No. 6 shows early signs of microstructural deterioration due to the accumulation of high local shear and nonuniform stress. The probable cause of inadequate lubrication was that the frictional growth, high contact stresses, and grain boundary and micro-voids were enhanced. Even though the gross bending angle may still be acceptable, subsurface integrity is severely degraded.

The sample with reduced BDC (Combination No. 13) showed more marked surface disturbances on its outer and internal radii. The outer radius showed local surface cracking and rougher topography, while the inner radius presented heterogeneous deformation features and areas of surface disruption. These results correspond to poor through-thickness material compression during bending, leading to localized strain accumulation at the surface. Grain-scale separation cannot be confirmed from the SEM images, but surface cracking and a roughened morphology suggest that formation stability is lower at lower injection levels.

Combination No. 14 showed surface attributes intermediate between the reference and Combination No. 13. Friction-related markings and surface irregularities were observed, especially within the inner radius; however, the overall surface consistency appeared better than in the reduced-injection condition. The deformation behavior was more uniform across the observed region, and surface cracks were less frequent. This indicates that an adequate injection depth leads to more uniform macroscopic deformation than a non-friction-rich surface area, even in unfavorable lubrication environments. However, higher friction was observed at the tool–material interface, as evidenced by surface scoring and site-specific adhesion marks.

The comparison of these samples indicates a strong dependence of microstructural quality in bent regions on BDC depth, even under identical wear and lubrication conditions. The low BDC condition (combination No. 13) caused the under-compressed inner zone of grain decohesion and surface fracturing, whereas the optimum one (combination No. 14) contributed to better stress redistribution and smoother grain deformation. Microstructural information is consistent with mechanical measurements, and a higher BDC weight ratio leads to greater bending in all bending cases. Both cases, however, show increased friction-induced surface damage and strain heterogeneity in the presence of lubrication, as evidenced by bright frictional ridges and deformation bands present in the inner radius.

In general, SEM observation validates that, although tribological phenomena and punch wear control surface finish and microstructural integrity are important, it is the BDC level that governs the extent of plastic accommodation, determining whether deformation will remain ductile or rapidly transform into brittle failure. The interaction between the worn tool and poor lubrication further accentuates stress localization and promotes premature microcrack initiation—situations that may, under metal forming in long-term production campaigns, eventually hasten fatigue failure and form instability.

It results from material cracking or decohesion along grain boundaries, which can lead to grain detachment. It often happens due to weak grain boundaries, impurities, corrosion, or creep. Grain boundary separation could result in reduced ductility, crack initiation sites, loss of strength, surface degradation, and accelerated corrosion. All listed effects are important for the further use of the part and its operation.

Even if in the reference sample, clear material cracks are hard to notice; they appear in all other samples, especially on the inner radius. The best example would be combination No. 13, where not only cracks but also clear material crumbling could be observed. Between combinations No. 13 and 14, only the BDC level differed; however, the tests were performed so that the BDC was reduced rather than increased, which indicated that such a part-edge difference should be observed. As the BDC level is reduced, the material is less compressed, and fewer defects should occur. This part-edge difference between samples could be explained by the two following points:

1. There is a possibility that such a sample happened by chance, and if more parts were inspected for combinations No. 6 and 14, similar results could be acquired.
2. Because of the BDC level difference, it is possible that compressive stress force is sufficient for the edge material to crumble, but insufficient for material extrusion, as in combinations No. 6 and 14.

Between combinations No. 6 and 14, the only parameter that differed was lubrication. The lubrication effect was clearly visible in the inner radius photos. Combination No. 14 edges of the part have brighter, sharper edges, which are formed when a material, due to frictional forces, sticks to the bending matrix and is deformed, resulting in excess material being pushed out. Lubrication reduces friction as well as reduces shear stress on the material surface, resulting in deformation that is more homogeneous across the section, preventing excessive strain concentration near the tool contact areas, resulting in grain deformation becoming more uniform through the workpiece thickness and allowing inner grains in the compressive zone, thus maintaining relatively equiaxed or slightly compressed shapes rather than showing severe buckling or folding. While these observations suggest trends in localized surface deformation, the available data are insufficient to draw definitive conclusions regarding bulk grain structure or metallurgical transformations.

Across all examined samples, the appearance of SEM differences varied mainly between analyses by surface roughness, shear markings, and surface discontinuities. These details were characteristic of changes in near-surface deformation behavior that was induced by process conditions such as BDC, clearance, tool state, and lubrication. However, SEM analysis does not provide enough evidence to make direct comparisons between samples with respect to grain form, grain deformation, or dislocation activity. Thus, all conclusions on crystallographic distortion mechanisms, intergranular decohesion, or dislocation-based plasticity are out of the scope of this present investigation. Trends in surface morphology qualitatively match the dimensional measurements obtained by the CMM. Process conditions with greater dimensional deviation exhibit more severe surface irregularities and localized deformation features, suggesting a link between geometric fidelity and surface integrity in progressive stamping.

## 4. Conclusions

This research indicated a relationship between the effects of stamping process variables and par quality. After conducting research, the following conclusions were formulated:

1. The BDC level of the tool has the greatest influence on the bending angles of the part (BDC influence was 181 on angle AB and 172 for angle AC). Optimal BDC ensures uniform through-thickness flow and minimizes elastic recovery, while BDC produces nonuniform stresses that promote localized thinning and folding.
2. Die and punch dimensional clearance strongly affects accuracy (clearance influence was 102 on angle AB and 126 for angle AC). Smaller clearance changes significantly alter local bending stresses.
3. In the scope of this study, tool condition and lubrication showed a secondary influence on measured bending angles (wear influence was 36 on angle AB and 11 for angle AC, and lubrication influence was 11 on angle AB and 2 for angle AC), but the SEM observations suggest that these factors affect surface morphology and near-surface damage of the stamped parts.
4. Process variables act interactively rather than independently. Parameter coupling, especially between BDC and wear (the combined influence was 32 on angle AB and 16 on angle AC), governs process stability, indicating that single-factor optimization cannot sustain part quality without compensating for changes in clearance and lubrication.
5. Tribological conditions remain essential for reliability. Even if not the main source of bend angle deviation, poor lubrication reduces dislocation density, increasing susceptibility to fatigue and corrosion.
6. The combined CMM and SEM methodology is reproducible and effective. Correlating geometric deviations with microstructural response provides a strong basis for the development of digital twins and data-driven prediction models for progressive stamping.

## Figures and Tables

**Table 1:** S355MC material, used for tests and mechanical properties.

Properties	Value
Yield strength (*R_e_*), MPa	451.2
Tensile strength (*R_m_*), MPa	510.1
Elongation (*A*), %	42.3

**Table 2:** Essential parameters of the bending process and their levels.

Process Parameter	Marking	High Level (+)	Low level (−)
Bottom dead center	A	339.1 mm	338.8 mm
Clearance	B	1.0 mm	0.8 mm
Punch condition	C	New (R0.0 mm)	Worn out (R0.1 mm)
Lubrication	D	Enabled (6 g/m^2^)	Off (0 g/m^2^)

**Table 3:** Combinations of the factors possible.

Combination No.	A	B	C	D
1	−	−	+	+
2	+	−	+	+
3	−	+	+	+
4	+	+	+	+
5	−	−	−	+
6	+	−	−	+
7	−	+	−	+
8	+	+	−	+
9	−	−	+	−
10	+	−	+	−
11	−	+	+	−
12	+	+	+	−
13	−	−	−	−
14	+	−	−	−
15	−	+	−	−
16	+	+	−	−

**Table 4:** Test data and results.

Test Execution Sequence	Combination No.	Test Group	Bottom Dead Center Level, mm	Clearance, mm	Punch Radius, mm	Lubrication	Angle, °	
							*AB*	*AC*
1	1	1	338.8	0.8	R0.0	0 g/m^2^	89.287	88.806
2	1	1	338.8	0.8	R0.0	0 g/m^2^	89.141	88.762
3	1	1	338.8	0.8	R0.0	0 g/m^2^	89.192	88.787
4	1	1	338.8	0.8	R0.0	0 g/m^2^	89.185	88.798
5	1	1	338.8	0.8	R0.0	0 g/m^2^	89.193	88.755
6	2	1	339.1	0.8	R0.0	0 g/m^2^	90.963	91.061
7	2	1	339.1	0.8	R0.0	0 g/m^2^	90.976	91.023
8	2	1	339.1	0.8	R0.0	0 g/m^2^	91.009	91.064
9	2	1	339.1	0.8	R0.0	0 g/m^2^	91.027	91.088
10	2	1	339.1	0.8	R0.0	0 g/m^2^	91.011	91.083
11	3	1	338.8	1.0	R0.0	0 g/m^2^	90.445	90.476
12	3	1	338.8	1.0	R0.0	0 g/m^2^	90.409	90.959
13	3	1	338.8	1.0	R0.0	0 g/m^2^	90.436	90.757
14	3	1	338.8	1.0	R0.0	0 g/m^2^	90.507	90.772
15	3	1	338.8	1.0	R0.0	0 g/m^2^	90.416	90.555
16	4	1	338.8	1.0	R0.0	6 g/m^2^	90.655	90.485
17	4	1	338.8	1.0	R0.0	6 g/m^2^	90.389	90.551
18	4	1	338.8	1.0	R0.0	6 g/m^2^	90.219	90.373
19	4	1	338.8	1.0	R0.0	6 g/m^2^	90.372	90.397
20	4	1	338.8	1.0	R0.0	6 g/m^2^	90.335	90.373
21	5	1	339.1	1.0	R0.0	0 g/m^2^	92.326	93.292
22	5	1	339.1	1.0	R0.0	0 g/m^2^	92.367	93.206
23	5	1	339.1	1.0	R0.0	0 g/m^2^	92.375	93.242
24	5	1	339.1	1.0	R0.0	0 g/m^2^	92.423	93.149
25	5	1	339.1	1.0	R0.0	0 g/m^2^	92.366	93.115
26	6	1	339.1	1.0	R0.0	6 g/m^2^	92.264	93.342
27	6	1	339.1	1.0	R0.0	6 g/m^2^	92.296	93.182
28	6	1	339.1	1.0	R0.0	6 g/m^2^	92.343	93.138
29	6	1	339.1	1.0	R0.0	6 g/m^2^	92.330	93.118
30	6	1	339.1	1.0	R0.0	6 g/m^2^	92.317	93.002
31	7	1	339.1	0.8	R0.0	6 g/m^2^	91.112	91.154
32	7	1	339.1	0.8	R0.0	6 g/m^2^	91.104	91.122
33	7	1	339.1	0.8	R0.0	6 g/m^2^	91.117	91.183
34	7	1	339.1	0.8	R0.0	6 g/m^2^	91.085	91.147
35	7	1	339.1	0.8	R0.0	6 g/m^2^	91.102	91.139
36	8	1	338.8	0.8	R0.0	6 g/m^2^	89.077	88.715
37	8	1	338.8	0.8	R0.0	6 g/m^2^	89.076	88.720
38	8	1	338.8	0.8	R0.0	6 g/m^2^	89.102	88.733
39	8	1	338.8	0.8	R0.0	6 g/m^2^	89.042	88.710
40	8	1	338.8	0.8	R0.0	6 g/m^2^	89.087	88.696
41	9	2	338.8	0.8	R0.1	6 g/m^2^	88.417	88.519
42	9	2	338.8	0.8	R0.1	6 g/m^2^	88.421	88.504
43	9	2	338.8	0.8	R0.1	6 g/m^2^	88.408	88.521
44	9	2	338.8	0.8	R0.1	6 g/m^2^	88.437	88.571
45	9	2	338.8	0.8	R0.1	6 g/m^2^	88.468	88.485
46	10	2	339.1	0.8	R0.1	6 g/m^2^	90.990	91.184
47	10	2	339.1	0.8	R0.1	6 g/m^2^	91.079	91.245
48	10	2	339.1	0.8	R0.1	6 g/m^2^	91.045	91.195
49	10	2	339.1	0.8	R0.1	6 g/m^2^	91.037	91.229
50	10	2	339.1	0.8	R0.1	6 g/m^2^	91.011	91.182
51	11	2	339.1	0.8	R0.1	0 g/m^2^	90.943	91.172
52	11	2	339.1	0.8	R0.1	0 g/m^2^	90.997	91.183
53	11	2	339.1	0.8	R0.1	0 g/m^2^	91.004	91.189
54	11	2	339.1	0.8	R0.1	0 g/m^2^	90.923	91.119
55	11	2	339.1	0.8	R0.1	0 g/m^2^	90.950	91.171
56	12	2	338.8	0.8	R0.1	0 g/m^2^	88.502	88.527
57	12	2	338.8	0.8	R0.1	0 g/m^2^	88.419	88.539
58	12	2	338.8	0.8	R0.1	0 g/m^2^	88.469	88.558
59	12	2	338.8	0.8	R0.1	0 g/m^2^	88.472	88.564
60	12	2	338.8	0.8	R0.1	0 g/m^2^	88.445	88.553
61	13	2	338.8	1.0	R0.1	0 g/m^2^	89.117	90.003
62	13	2	338.8	1.0	R0.1	0 g/m^2^	89.274	90.019
63	13	2	338.8	1.0	R0.1	0 g/m^2^	89.259	90.036
64	13	2	338.8	1.0	R0.1	0 g/m^2^	89.242	90.063
65	13	2	338.8	1.0	R0.1	0 g/m^2^	89.237	90.055
66	14	2	339.1	1.0	R0.1	0 g/m^2^	92.257	93.299
67	14	2	339.1	1.0	R0.1	0 g/m^2^	92.241	93.274
68	14	2	339.1	1.0	R0.1	0 g/m^2^	92.241	93.343
69	14	2	339.1	1.0	R0.1	0 g/m^2^	92.288	93.307
70	14	2	339.1	1.0	R0.1	0 g/m^2^	92.239	93.335
71	15	2	339.1	1.0	R0.1	6 g/m^2^	92.262	93.216
72	15	2	339.1	1.0	R0.1	6 g/m^2^	92.287	93.285
73	15	2	339.1	1.0	R0.1	6 g/m^2^	92.268	93.209
74	15	2	339.1	1.0	R0.1	6 g/m^2^	92.285	93.230
75	15	2	339.1	1.0	R0.1	6 g/m^2^	92.245	93.254
76	16	2	338.8	1.0	R0.1	6 g/m^2^	89.805	90.552
77	16	2	338.8	1.0	R0.1	6 g/m^2^	89.873	90.604
78	16	2	338.8	1.0	R0.1	6 g/m^2^	89.751	90.506
79	16	2	338.8	1.0	R0.1	6 g/m^2^	89.825	90.594
80	16	2	338.8	1.0	R0.1	6 g/m^2^	89.913	90.627

**Table 5:** Microscope scanning results.

Combination No.	Outer Radius	Inner Radius
4	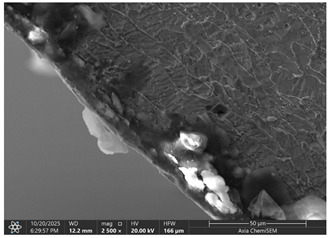	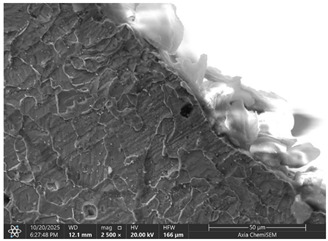
6	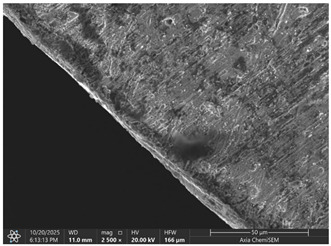	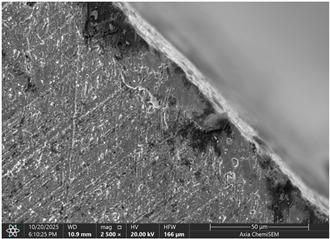
13	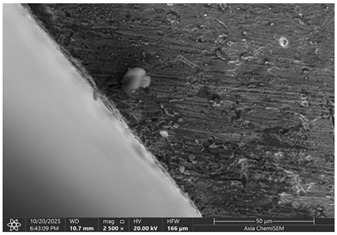	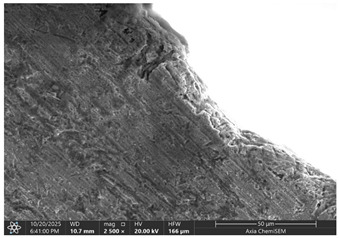
14	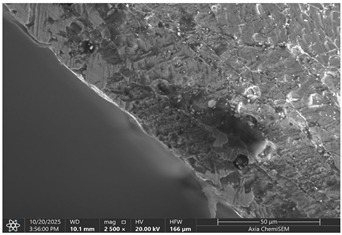	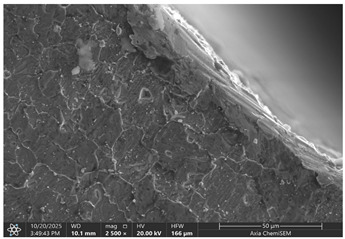

## Data Availability

The original contributions presented in this study are included in the article. Further inquiries can be directed to the corresponding author.

## References

[1] Molitor D.A., Kokozinski A., Kubik C., Arne V., Veitenheimer C., Georgi F., Krämer R., Groche P. (2025). Identifying productivity-limiting factors in progressive die stamping: Data-driven methodology for process optimization. Prod. Eng..

[2] Dwi R., Abroor H., Fauzi M., Pradhana H.B. (2025). The Influence of Progressive Die Design on Productivity and Quality in Metal Stamping Processes. SSRN Electron. J..

[3] Farioli D., Kaya E., Fumagalli A., Cattaneo P., Strano M. (2023). A Data-Based Tool Failure Prevention Approach in Progressive Die Stamping. J. Manuf. Mater. Process..

[4] Rizk J., Rachik M., Maillard A. (2024). Finite element simulation of the complete sheet metal blanking cycle: Effect of blanking clearance on force curve and cut edge quality. Heliyon.

[5] Mucha J., Tutak J. (2019). Analysis of the Influence of Blanking Clearance on the Wear of the Punch, the Change of the Burr Size and the Geometry of the Hook Blanked in the Hardened Steel Sheet. Materials.

[6] Chan H.Y., Abdullah A.B. (2014). Geometrical Defect in Precision Blanking/Punching: A Comprehensive Review on Burr Formation. Res. J. Appl. Sci. Eng. Technol..

[7] Lal R.K., Choubey V.K., Dwivedi J.P., Kumar S. (2018). Study of factors affecting Springback in Sheet Metal Forming and Deep Drawing Process. Mater. Today Proc..

[8] Trzepieciński T. (2023). Approaches for Preventing Tool Wear in Sheet Metal Forming Processes. Machines.

[9] Kubik C., Becker M., Molitor D.-A., Groche P. (2022). Towards a systematic approach for wear detection in sheet metal forming using machine learning. Prod. Eng..

[10] Molitor D.A., Kubik C., Hetfleisch R.H., Groche P. (2022). Workpiece image-based tool wear classification in blanking processes using deep convolutional neural networks. Prod. Eng. Res. Devel..

[11] Schlegel C., Molitor D.A., Kubik C., Martin D.M., Groche P. (2023). Tool wear segmentation in blanking processes with fully convolutional networks based on digital image processing. J. Mater. Process. Technol..

[12] Ahmed G.M.S., Ahmed H., Mohiuddin M.V., Sajid S.M.S. (2014). Experimental Evaluation of Springback in Mild Steel and its Validation Using LS-DYNA. Procedia Mater. Sci..

[13] Chandrasekharan S., Palaniswamy H., Jain N., Ngaile G., Altan T. (2005). Evaluation of stamping lubricants at various temperature levels using the ironing test. Int. J. Mach. Tools Manuf..

[14] Folle L.F., Lima T.N., Santos M.P.S., Callegari B., Silva B.C.d.S., Zamorano L.G.S., Coelho R.S. (2024). A Review on Sheet Metal Forming Behavior in High-Strength Steels and the Use of Numerical Simulations. Metals.

[15] Shubham M., Dinesh S., Chavan G. (2025). A Literature Review On Optimization of Stamping Tool. Int. J. Emerg. Technol. Innov. Res..

[16] Guo F., Jeong H., Park D., Kim G., Sung B., Kim N. (2024). Numerical Optimization of Variable Blank Holder Force Trajectories in Stamping Process for Multi-Defect Reduction. Materials.

[17] Ji W., Bai Y. (2025). Concurrent shape and topology optimization of stamping thin-walled structures based on density filtering method. Comput. Methods Appl. Mech. Eng..

[18] Zhang H., Wei W., Long S., Zhou M., Li C. (2025). Optimization of Stamping Process Parameters for Sustainable Manufacturing: Numerical Simulation Based on AutoForm. Sustainability.

[19] Vrh M., Halilovič M., Starman B., Štok B. (2009). Modelling of springback in sheet metal forming. Int. J. Mater. Form..

[20] Wang Y., Zhong Q., Hua R., Cheng L., Wang C., He H., Chen F., Ma Z. (2023). Ultrasonic Vibration-Assisted Stamping of Serpentine Micro-Channel for Titanium Bipolar Plates Used in Proton-Exchange Membrane Fuel Cell. Materials.

